# Palliative Care for Patients With Breast Cancer in a Palliative Care Unit of a Japanese Hospital

**DOI:** 10.7759/cureus.67834

**Published:** 2024-08-26

**Authors:** Bunzo Nakata, Chie Sakimura, Masashige Tendo, Tomoya Tsukida, Takeshi Hori, Kosei Hirakawa, Tetsuro Ishikawa

**Affiliations:** 1 Surgery, Kashiwara Municipal Hospital, Kashiwara, JPN

**Keywords:** sedation, hypercalcemia, disseminated intravascular coagulation, jaundice, pleural effusion, breast cancer

## Abstract

Background

This study describes the end-of-life care management in palliative care units (PCUs) for patients with breast cancer in Japan.

Methods

The medical data of patients admitted to the palliative care unit of Kashiwara Municipal Hospital between October 2017 and December 2023 were analyzed. A chi-square test was conducted to analyze the data using the Excel software (Microsoft Corp., Redmond, WA).

Results

The most common clinical condition among the 32 patients with breast cancer in our palliative care unit was pleural effusion (17/32, 53.1%), followed by obstructive jaundice (6/32, 18.8%), disseminated intravascular coagulation (DIC) (4/32, 12.5%), and hypercalcemia (1/32, 3.1%). Most patients had no indications for pleural effusion removal, biliary drainage, or anticoagulation therapy. Palliative sedation was performed in 25% of the patients with breast cancer, mainly to relieve intolerable general fatigue. There were no statistically significant differences in the sedation ratios between breast cancer and cancers at other primary sites.

Conclusion

Palliative treatments using appropriate infusion, narcotics, oxygen administration, various drugs, and sedation were administered in our palliative care unit to relieve symptoms instead of radical treatments for severe clinical conditions of breast cancer.

## Introduction

Breast cancer is the second leading cause of cancer-related death among females in the United States (after lung cancer). The number of female deaths due to breast cancer in 2024 is estimated to be 42,250 in the United States [1]. In Japan, breast cancer is the fourth leading cause of cancer-related death in females (after colon, lung, and pancreatic cancers). The number of female deaths due to breast cancer in Japan by 2022 is estimated to be 15,912 [2].

Palliative care in Japan has rapidly progressed over the last decade. In 2013, Morita and Kizawa reported 215 palliative care units (PCUs) in Japan [3]. In 2020, the Japanese Ministry of Health, Labour and Welfare established PCUs in 479 general hospitals (6.7% of the total number of general hospitals), consisting of 9,498 beds [4].

Although the Japanese government has been attempting to develop more efficient home care services for terminal-stage cancer patients, at times, such patients need to be treated in a PCU. In this study, the details of end-of-life care for patients with breast cancer in the PCU of a medium-sized hospital in Japan are presented.

Most clinical studies on breast cancer have focused on curative treatment with very few focusing on the palliative treatment of terminal breast cancer. Therefore, our study is aimed at sharing knowledge that could aid other palliative care units in alleviating patient symptoms.

## Materials and methods

Patients

The PCU at Kashiwara Municipal Hospital was established in October 2017. As of December 31, 2023, 1,080 patients with various cancers had been admitted to the PCU. In this study, clinical data from the medical records of patients who were charged by one doctor (BN) certified by the Japanese Society for Palliative Medicine were used to omit doctor bias. During this period, the doctor treated 460 patients, including 32 with breast cancers, 143 with gastrointestinal cancer, 109 with hepatobiliary and pancreatic cancers, 97 with lung cancer, 25 with head and neck cancer, and 54 with miscellaneous cancers. Triple-negative breast cancer (TNBC) has no targetable molecules for treatment, including estrogen receptor, progesterone receptor, and human epidermal growth factor receptor 2 (HER2). Jaundice was defined as a serum bilirubin level of ≥3 mg/dL, because patients above this value usually exhibited clinically evident jaundice through findings of yellowish skin and/or conjunctival membranes over the sclerae due to hyperbilirubinemia. Disseminated intravascular coagulation (DIC) was determined using the International Society on Thrombosis and Haemostasis (ISTH) DIC scoring system. Hypercalcemia was defined as a total serum calcium concentration of >10.5 mg/dL. Symptoms that the patients expressed verbally were counted from the medical records.

Statistical analysis procedure

Statistical analysis was performed with chi-square tests to compare the number of sedated and non-sedated patients with breast cancer and other malignancies using the Excel software (Microsoft Office LTSC Standard 2021, Microsoft Corp., Redmond, WA). P values of <0.05 were considered significant.

Ethical approval

This study was approved by the Ethics Committee of Kashiwara Municipal Hospital (approval number: 2024-2). The opt-out document was posted in the outpatient waiting room and wards of Kashiwara Municipal Hospital, in which the patients were assured of their right to refuse and to withdraw from the study at any stage.

## Results

Treatment prior to admission to the PCU

The median age of the patients with breast cancer examined in this study was 69 years (ranging from 28 to 92 years).

Table 1 shows the surgery for the primary tumor and treatments for recurrent tumors prior to admission to the PCU. All but two patients underwent surgical treatment for primary tumors, including mastectomy or partial mastectomy with sentinel lymph node biopsy or axillary lymph node resection.

**Table 1:** Treatments for patients with breast cancer prior to admission to the palliative care unit Number in parenthesis: percentage to all patients CDK4/6, cyclin-dependent kinase 4/6; HER2, human epidermal growth factor receptor 2

Characteristics	Values
Number of patients	32 (100%)
Number of patients who underwent surgical treatment for primary tumors	30 (93.8%)
Number of patients who underwent individual treatments for recurrent tumors	
Resection for metastatic tumor	7 (21.9%)
Radiation therapy	18 (56.3%)
Chemotherapy	21 (65.6%)
Hormonotherapy	14 (43.8%)
CDK4/6 inhibitor (palbociclib and abemaciclib)	7 (21.9%)
Anti-HER2 therapy	3 (9.4%)
Bone-modifying agents (zoledronic acid and denosumab)	6 (18.8%)

Various treatments were administered after recurrence. Resections for recurrent tumors were performed on seven patients: the same side mastectomy for one patient after partial mastectomy, resections of skin metastases for two patients, the resection of thoracic wall metastasis for one patient, resections of axillary lymph node metastases for two patients, and the resection of brain metastasis for one patient.

More than half of the patients underwent irradiation. One patient was independently irradiated in the bone, brain, and lymph nodes. Other patients were irradiated in one area individually: the bone for nine patients, the brain for five patients, lymph nodes for two patients, and the spinal cord for one patient.

Metastatic sites, clinical conditions, symptoms, and palliative treatments

Table 2 shows the general conditions of the patients with breast cancer and their treatments in our PCU.

**Table 2:** Clinicopathologic features and treatment for patients with breast cancer in the palliative care unit

Characteristics	Number of patients (percentage to all patients)
All patients	32 (100%)
Number of patients with triple-negative breast cancer	9 (28.1%)
Number of patients with individual metastatic site	
Bone	18 (56.3%)
Lung	17 (53.1%)
Lymph node	14 (43.8%)
Liver	11 (34.4%)
Brain	8 (25%)
Skin	4 (12.5%)
Meninges	3 (9.4%)
Number of patients developing individual clinical conditions	
Pleural effusion	17 (53.1%)
Jaundice	6 (18.8%)
Disseminated intravascular coagulation	4 (12.5%)
Hypercalcemia	1 (3.1%)
Symptom	
Anorexia	32 (100%)
Pain	23 (71.9%)
General fatigue	20 (62.5%)
Constipation	20 (62.5%)
Dyspnea	17 (53.1%)
Somnolence	12 (37.5%)
Insomnia	11 (34.4)
Nausea	9 (28.1%)
Cough	8 (25%)
Delirium	4 (12.5%)
Palliative treatment	
Infusion	32 (100%)
Peripheral intravenous infusion	17 (53.1%)
Infusion via central intravenous catheter	12 (37.5%)
Continuous subcutaneous infusion	3 (9.4%)
Narcotics	26 (81.3%)
Fentanyl skin patch	17 (53.1%)
Continuous intravenous narcotics	7 (21.9%)
Oral narcotics	2 (6.25%)
Oxygen administration	19 (59.4%)
Steroid	16 (50%)
Sleeping pill	15 (46.9%)
Antidiuretic agent	7 (21.9%)
Pleural effusion removal	3 (9.4%)

More than half of the patients developed bone and/or lung metastases. Metastases to the lymph nodes and liver were frequently observed. Brain metastases occurred in one-quarter of the patients.

More than half the patients had pleural effusion, which was diagnosed using plain chest radiography and/or plain chest computed tomography. Pleural effusion was removed in only three patients. Pleural effusion drainage was continued for five, six, and 18 days, until their date of death. The total drainage volumes were 5,200 mL, 1,550 mL, and 5,600 mL, respectively.

The cause of jaundice was biliary obstruction due to multiple hepatic metastases, except in one patient with hepatic failure due to DIC. Biliary drainage was not performed because of biliary obstruction.

Four patients developed cancer-associated DIC. Unfractionated heparin was given to one patient; however, she died two days after the DIC diagnosis. The other three patients with DIC were not administered anti-DIC agents and died five, six, and 13 days after DIC diagnosis. No platelet transfusions, fresh frozen plasmas, or antithrombotic agents/thrombomodulin were given to patients with DIC.

One patient with multiple bone metastases developed cancer-related hypercalcemia (maximum: 14.3 mg/dL) and was treated with calcitonin for 10 days, giving no other managements for hypercalcemia such as hydration or bisphosphonates; however, she died 34 days after being diagnosed with hypercalcemia.

The most common symptoms are anorexia, pain, general fatigue, and constipation. Infusions were administered to all patients to relieve dehydration. Peripheral intravenous infusions were administered to approximately half of all patients, of whom three were placed with a peripherally inserted central venous catheter (PICC) due to difficulty in securing the vein. The infusion amount per day was 1,000 mL for four patients and 500 mL for 13 patients. The infusion duration was 5-23 days (median: 10 days). Total parenteral nutrition (TPN) was provided for approximately one-third of the patients, of whom five received PICC, three received central venous (CV) access devices (CV ports) while in the PCU, and four had implanted CV ports for chemotherapy prior to admission to the PCU. The infusion volume per day was 720 mL to 1,000 mL. The duration of total parenteral nutrition was one to 54 days (median: seven days). Continuous subcutaneous infusion was administered to three patients. The daily infusion volume was 500 mL. The durations of infusion were two, three, and 33 days.

The patients experienced pain in various parts of their bodies. Four patients reported experiencing pain in their entire bodies. Nineteen patients complained of one to three pain areas: head, two; neck, two; upper limb, one; shoulder, two; chest, two; abdomen, two; lower back, seven; and lower limb, four. To relieve pain, fentanyl skin patches were attached to about half of all patients to control their pain. The ratio of fentanyl transdermal patch use to all narcotic drugs used to relieve pain was 73.9% (17 of 23 patients with pain). Once-a-day fentanyl citrate patch (Fentos® tape, Hisamitsu Pharmaceutical Co., Inc., Saga, Japan) was used as a fentanyl transdermal patch. One milligram of the fentanyl transdermal patch is equivalent to 300 mcg/day of fentanyl injection. The amount of fentanyl transdermal patches administered was 0.5 mg to 4 mg (median: 2 mg). Oral narcotics were administered to only two patients because most patients complained of anorexia, and some of them had nausea and difficulty taking oral drugs. Metoclopramide or haloperidol injection was given to the patient who suffered from nausea. Among the seven patients administered continuous intravenous narcotics, three patients had no pain or dyspnea, for which low doses of morphine (12-24 mg per day) were administered.

Sedation

Eight patients with breast cancer were sedated using midazolam (Table 3). The chief complaints requiring sedation were general fatigue in seven patients and insomnia due to a severe cough in one patient. The duration from admission to sedation was six to 87 days (median: 22 days). The duration of sedation was one to 12 days (median: two days). The maximum dose of midazolam for individual patients was 0.5 mg to 2 mg per hour (median: 1.5 mg per hour); at those doses, the patients were deeply sedated, evaluated by Richmond Agitation-Sedation Scale (RASS): -4 to -5.

**Table 3:** Sedated patient ratio in the respective primary tumor site *Tested by chi-square test for the sedated rate of individual cancer compared to that of breast cancer †Thirteen origin unknown cancers, 11 orthopedic malignant tumors, nine skin cancers, seven hematological malignancies, four central nerve system malignancies, three malignant mesothelioma, two adrenal malignant tumors, one bladder cancer, one ovarian cancer, one malignant thymoma, one neuroendocrine tumor, and one malignant cardiac tumor

Primary tumor	Total (n=406)	Sedated (n=90)	Non-sedated (n=316)	P value^*^
Breast cancer	32	8 (25%)	24 (75%)	-
Gastrointestinal cancer	143	32 (22.4%)	111 (77.6%)	0.7495
Hepatobiliary and pancreatic cancers	109	19 (17.4%)	90 (82.6%)	0.3387
Lung cancer	97	13 (13.4%)	84 (86.6%)	0.1233
Head and neck cancers	25	10 (40%)	15 (60%)	0.2267
Miscellaneous cancers^†^	54	7 (13%)	47 (87%)	0.1551

The percentage of sedated patients with individual primary tumor sites was not significantly different from that of patients with breast cancer (Table 3).

## Discussion

Table 1 shows that the breast cancers examined in our PCU were treated with multidisciplinary treatments, as is generally performed in clinical practice [5].

TNBC accounts for 10%-20% of invasive breast cancers and is characterized by a poorer prognosis than other breast cancer subtypes [6]. The incidence of TNBC in the patients examined in this study was 28.1%, which was not significantly different from that in the general population (Table 2).

Metastasis in various organs can lead to severe clinical conditions (Table 2). The most frequent clinical condition among patients was pleural effusion. Breast cancer is the second leading cause of malignant pleural effusion (MPE), after lung cancer [7]. Current treatments for MPE are palliative and include drainage via thoracentesis, pleurodesis, and pleuroperitoneal shunting [8]. Generally, treatment options for MPE are selected, referring to the patient’s prognosis, preference, functional status, rate of pleural effusion accumulation, and so forth [8]. In our PCU, the majority of patients with pleural effusion were treated with oxygen and/or narcotics, such as morphine, without pleural effusion removal, considering the poor prognosis and severe conditions of the patients.

The present cases of jaundice were caused by massive liver metastases; therefore, there were no indications for biliary drainage in these patients. However, in a rare case of obstructive jaundice due to lymph node metastasis to the hepatoduodenal ligament, the patient was successfully treated with endoscopic retrospective biliary drainage [9]. In a rarer group of patients whose jaundice was caused by the obstruction of the extrahepatic bile ducts by metastases to the bile duct, ampulla, or head of the pancreas, relief from biliary obstruction using surgical bypass or biliary stenting may extend their survival [10].

The treatment of DIC in patients with solid tumors remains controversial based on anecdotal reports and published recommendations achieved by consensus. Sakuragawa et al. conducted a clinical multi-institutional trial and demonstrated that both unfractionated heparin and low-molecular-weight heparin improved DIC status in most patients with solid tumors [11]. One patient with DIC in our PCU was treated with unfractionated heparin; however, their DIC status did not improve. The principle of DIC treatment is to eliminate its causes. Consequently, in cancer-associated DIC, specific chemotherapy should be initiated if it is tolerated by the patient [12]. However, none of the patients with DIC in this study tolerated chemotherapy. Under these conditions, a useful treatment for DIC is not expected.

The main mechanism of hypercalcemia in patients with malignant tumors is the excessive production of parathyroid hormone-related peptides. Local osteolytic hypercalcemia is the most prevalent cause of hypercalcemia in breast cancer patients with bone metastases [13]. Hydration is the initial step in hypercalcemia to relieve symptoms in a dehydrated state; however, it does not substitute for curative treatment with bisphosphonates such as zoledronic acid and calcitonin, which are adjunctive agents for patients with severe hypercalcemia owing to their rapid action [14]. Despite the current availability of more effective treatments, outcomes remain poor, with a median survival of 25-52 days after the onset of hypercalcemia [15]. A patient treated with calcitonin in our PCU had short survival.

Regarding symptoms and palliative treatments in our PCU, the following two points were especially important: infusion access and the selection of narcotics. A PICC was inserted in eight of the 32 patients during their stay in the PCU because many types of injective agents are available. The effect of TPN for patients with terminal malignancy has not been fully elucidated. In this series, the durations of TPN were short (median: seven days), suggesting that the benefits of TPN were questionable for the breast cancer patients in the PCU. Fentanyl transdermal patches were administered for pain control in more than half of the patients because of difficulty in oral narcotic intakes. These treatment options should be altered according to the general condition and prognosis of the patients.

Palliative sedation should be considered in patients with intolerable symptoms due to cancer [16]. The frequency of sedation for patients with terminal malignancies ranges from 1% to approximately 50%, depending on physicians’ experiences, and is influenced by their cultural, religious, and ethical backgrounds [17]. In our PCU, the frequency of sedation for patients with breast cancer was not significantly different from that for patients with malignancies at individual primary sites under conditions of palliative sedation induced by the same physician.

The patients with breast cancer examined here were deeply sedated at relatively low doses of midazolam alone. However, several patients with other malignancies in this series needed more than 5 mg per hour of midazolam plus a low dose of flunitrazepam. The different tolerance to midazolam might be related to the individual metabolism of this agent.

Although there has been no medical law about palliative sedation in Japan, the Japanese Society for Palliative Medicine demonstrates their guidelines for palliative sedation in Japanese language on their home page. We have obeyed these guidelines.

## Conclusions

Patients with breast cancer in the PCU develop various severe clinical conditions such as pleural effusion, jaundice, DIC, and hypercalcemia. The target of palliative treatments is to relieve symptoms and avoid radically curing these clinical conditions. An appropriate infusion route (peripheral vein, central intravenous catheter, or subcutaneous tissue) and narcotic administration method (skin patch, intravenous injection, or oral intake) should be considered carefully. It is a retrospective study with a limited number of terminal breast cancer patients; however, we trust that the information shared will contribute to the clinical dialogue and knowledge of treating terminally ill patients suffering from breast cancer.
